# Missing at random: a stochastic process perspective

**DOI:** 10.1093/biomet/asab002

**Published:** 2021-02-04

**Authors:** D. M. Farewell, R. M. Daniel, S. R. Seaman

**Affiliations:** Division of Population Medicine, School of Medicine, College of Biomedical and Life Sciences, Cardiff University, Cardiff CF14 4YS, U.K.; MRC Biostatistics Unit, University of Cambridge, Robinson Way, Cambridge CB2 0SR, U.K.

**Keywords:** Missingness at random, Sigma algebra, Stochastic process

## Abstract

We offer a natural and extensible measure-theoretic treatment of missingness at random. Within the standard missing-data framework, we give a novel characterization of the observed data as a stopping-set sigma algebra. We demonstrate that the usual missingness-at-random conditions are equivalent to requiring particular stochastic processes to be adapted to a set-indexed filtration. These measurability conditions ensure the usual factorization of likelihood ratios. We illustrate how the theory can be extended easily to incorporate explanatory variables, to describe longitudinal data in continuous time, and to admit more general coarsening of observations.

## 1 Introduction

Missing at random (Rubin, 1976) is a central concept in missing-data research. Nevertheless, recent papers (Seaman et al., 2013; Mealli & Rubin, 2015; Doretti et al., 2017) have argued that it remains poorly understood and often inaccurately articulated. The most common formulation (Little & Rubin, 2002, p. 12) is superficially intuitive, but misleading in its detail; accurate formulations exist (Robins & Gill, 1997; Lu & Copas, 2004), but typically hold little heuristic appeal.

For models under which data are missing at random, the likelihood is a product of two terms, a marginal likelihood and a conditional likelihood representing the missingness mechanism. By appealing to the theory of incompletely observed stochastic processes and characterizing these multiplicative components of the likelihood, we give a revealing proof of the likelihood factorization that depends only on simple measurability conditions. These conditions lead to a new, general definition of missingness at random that we find to be both rigorous and intuitive.

We represent data and all conditioning statements in terms of sigma algebras, principally to avoid any confusion over what information is being conditioned upon. As a byproduct, we obtain a single framework for discrete, continuous and more general random variables, whether partially observed in discrete time, in continuous time or under general forms of coarsening.

We tread this path with some trepidation. Rubin described his own initial measure-theoretic treatment of missing data as ‘window dressing’, and then-*Biometrika*-editor David Cox’s advice was to ‘eliminate all that measure theory noise’ (Lin et al., 2014). We hope our perspective avoids these pitfalls and exposes a signal that could be overlooked following such noise reduction.

## 2 Notation

Our starting point is a measurable space (Ω,*ℱ*) on which we define probability measures and random variables. We take a pure likelihood perspective (Royall, 1997) and compare just two candidate data-generating models. We aim to assess the evidence, as quantified by their likelihood ratio, in favour of a measure *P* relative to another measure *Q*. Since likelihood functions are just pairwise comparisons to an arbitrary reference measure, this simplification is not restrictive.

Following Pollard (2002, pp. 7–11), we adopt de Finetti notation: we allow the set *A* to also denote the indicator random variable 𝟙_*A*_, and reuse the symbol *P* to mean also its corresponding expectation operator *E_P_*, so that in particular *P*(*A*) = *P*(𝟙_*A*_) = *E_P_* (𝟙_*A*_) = ∫ 𝟙_*A*_ d*P*. One way of understanding this broader use of the symbol *P* is that we are extending its domain from indicator functions to more general random variables. In any event, the main consequence for this paper is that whenever we want to refer to *E_P_* we can simply write *P*.

The sigma algebra *ℱ* is a set of events that represents complete information about the entire stochastic system. In the present context, *ℱ* tells us the values of variables that may be observed or missing, and whether they are in fact missing. We avoid the term *complete data*, because its typical usage does not encompass indicators of missingness status, which in our view are certainly data. All available information we simply call *data* and represent by the sigma algebra *𝒟*. The data sigma algebra *𝒟* refers to as-yet-unrealized random variables, and contains all events whose logical status is known once the data are revealed. Until § 3 we remain nebulous about the precise definition of *𝒟* but, given our missing-data setting, *𝒟* will be a strict subset of *ℱ*. We write *σ*(*X*) to denote the sigma algebra generated by a random variable *X* ; *X* is measurable with respect to the sigma algebra *𝒟* if and only if *σ*(*X*) ⊆ *𝒟*.

We will assume whenever needed that probability measures are absolutely continuous with respect to one another. Then the customary measure-theoretic definition of the likelihood ratio comparing *P* and *Q* is the Radon–Nikodym derivative d*P*/d*Q* (Andersen et al., 1996, p. 97). Though somewhat formal in appearance, this object is simply a random variable that describes at each point in the sample space Ω the corresponding likelihood ratio of *P* relative to *Q*.

In general, the random variable d*P*/d*Q* will not be *𝒟*-measurable. This is because *P* and *Q* measure the size of each set in *ℱ* and, since *ℱ* ⊃ *𝒟*, the likelihood comparison d*P*/d*Q* may depend on events whose logical status cannot be determined solely from the data *𝒟*. In contrast, the likelihood ratio based on the data alone may be represented by its *𝒟*-measurable restriction

(1) $${\frac{\text{d}P}{\text{d}Q}|}_{D}=Q\left(\frac{\text{d}P}{\text{d}Q}|D\right).$$

This equality provides some intuition about the meaning of the left-hand side: the conditional expectation, given *𝒟* and with respect to *Q*, of the likelihood ratio d*P*/d*Q*. Loosely, this yields local averages of the random variable d*P*/d*Q* over each region of the sample space within which the data are constant.As noted by Chang & Pollard (1997, p. 299), this notation is more economical than standard representations such as *∫* *f* d*y*_mis_ because no *y*_mis_ need be introduced. Since (1) is data-measurable, it can be used for likelihood comparisons while satisfying standard chain rule relationships between likelihood ratios.

## 3 Monotone missing data

### 3.1 Data

Throughout this paper, we employ the machinery and methods of stochastic processes. For general missing data, the theory of stochastic processes indexed by sets will be required (Molchanov, 2006); however, we begin with the gentler case of monotone missingness, where it suffices to use standard theory for stochastic processes in discrete time. Unlike some other approaches, the stochastic process perspective permits ideas to be extended from the monotone case to the general setting by making essentially trivial semantic modifications.

Following Rubin (1976), we let *Y* = (*Y*_1_, …, *Y_n_*) be random variables defined on (Ω,*ℱ*), the ranges of which may be any measurable spaces. It is helpful to think of *Y* as a stochastic process *Y* = (*Y_m_*) indexed by discrete times *m* = 1, …, *n*. We observe *Y*_1_, …, *Y_M_*, where the integer-valued random variable *M* is also defined on (Ω,*ℱ*) and satisfies 0 ⩽ *M* ⩽ *n*. We do not observe *Y*_*M*+1_, …, *Y_n_*: observation of the stochastic process *Y* ceases at the random time *M*.

A filtration is a nested family of sigma algebras that captures the idea of information increase over time. When we say that a process (*Y_m_*) is adapted to a filtration (*𝒴_m_*), we mean that for each *m* the random variable *Y_m_* is measurable with respect to the sigma algebra *𝒴_m_*; adaptedness is just a sequence of measurability conditions. We define *𝒴_m_* = *σ*(*Y_l_* : *l* ⩽ *m*); the resulting (*𝒴_m_*) is called the natural filtration generated by *Y*, and by construction *Y* is adapted to (*𝒴_m_*).

The random variable *M* records the time at which observation of *Y* ceases, and is said to be a stopping time if at each point in time an observer knows whether observation of *Y* has already ceased. More specifically, *M* is a stopping time with respect to a filtration (*ℳ_m_*) if the event {*M* ⩽ *m*} belongs to *ℳ_m_* for all *m*. We can arrange for this to be the case by defining *ℳ_m_* = *σ*({*M* ⩽ *l*} : *l* ⩽ *m*). For each *m*, the sigma algebra *ℳ_m_* encodes logical information about whether the stopping event has occurred and, if so, at what point it occurred.

Until the stopping time *M*, we accrue information about both *Y* and *M*. We construct a larger filtration (*ℱ_m_*) by setting *ℱ_m_* = *𝒴_m_* ∨ *ℳ_m_*; the notation *𝒴_m_* ∨ *ℳ_m_* defines *ℱ_m_* as the smallest sigma algebra containing both *𝒴_m_* and *ℳ_m_*. Informally speaking, *ℱ_m_* tells us the values of *Y*_1_, …, *Y_m_* and, through knowledge of the indicators {*M* ⩽ 1}, …, {*M* ⩽ *m*}, if and when we have stopped recording measurements before time *m*. Recall that {*M* ⩽ *m*} denotes both the subset {*ω* : *M*(*ω*) ⩽ *m*} ⊆ Ω and the indicator random variable 𝟙_{*ω*:*M*(*ω*)⩽*m*}_.

Information increases until the random time *M*, at which point no further information is recorded. Since *M* is also a stopping time with respect to (*ℱ_m_*), this idea of information increase until a random time may be captured through the elegant definition of the stopping-time sigma algebra *ℱ_M_* = {*A* ∈ *ℱ* : *A* ∩ {*M* ⩽ *m*} ∈ *ℱ_m_* for all *m*} (Pollard, 2002, pp. 142–43). This *ℱ_M_* is precisely what we mean by the data sigma algebra *𝒟*; that is, we define *𝒟* = *ℱ_M_*. Despite being decorated with the letter *M*, we stress that the sigma algebra *ℱ_M_* is not itself a random object, but consists of that immutable set of events whose logical statuses are always known when the data are revealed, regardless of the particular realized values taken by *Y* or *M*.

For simplicity, we shall assume that *ℱ* = *ℱ_n_*, so that there are no measurable events beyond those described by *Y* and *M*. Similarly, we write *𝒴* = *𝒴_n_* or *ℳ* = *ℳ_n_* to describe complete information about *Y* or *M*, respectively. As a technical aside, we shall also assume that (Ω,*ℱ*) has a product structure that allows measures under which *𝒴* and *ℳ* are independent; that is, for all *A* ∈ *ℱ*, there exist *B* ∈ *𝒴* and *C* ∈ *ℳ* such that *A* = *BC*. In de Finetti notation, the product of events *BC* connotes 𝟙_*B*_𝟙_*C*_ = *B* ∩ *C*. Recall that two sigma algebras *𝒴* and *ℳ* are independent under *P* if and only if *P*(*BC*) = *P*(*B*)*P*(*C*) for all *B* ∈ *𝒴* and *C* ∈ *ℳ*.

It may be valuable at this point to consider the simplest possible example: *n* = 1 with a binary *Y*_1_ that could possibly be missing. In this case Ω is the four-point set {00, 01, 10, 11}, and *ℱ* = 2^Ω^ is the power set of Ω. For a generic element *bc* ∈ Ω, *Y*_1_(*bc*) = *b* and *M*(*bc*) = *c*. Then *𝒴*_0_ is the trivial sigma algebra *σ*(Ω) = {∅, Ω}, while *ℳ*_0_ = *ℱ*_0_ = *ℳ*_1_ = *ℳ* = *σ*({00, 10}, {01, 11}); *𝒴*_1_ = *𝒴* = *σ*({00, 01}, {10, 11}), and by assumption *ℱ*_1_ = *ℱ*. Rather less obviously, *𝒟* = *ℱ_M_* = *σ*({00, 10}, {01}, {11}). This makes intuitive sense, since when *M* = 0 we cannot hope to distinguish between *Y*_1_ = 0 and *Y*_1_ = 1. We can check our intuition more formally by verifying that the intersection of each of the atoms {00, 10}, {01} and {11} with {*M* ⩽ 0} = {00, 10} is in *ℱ*_0_. No finer partition is possible; for example, the singleton set {00}, when intersected with {*M* ⩽ 0} = {00, 10}, is not in *ℱ*_0_. Trivially, all intersections of the atoms {00, 10}, {01} and {11} with {*M* ⩽ 1} = Ω belong to *ℱ*_1_ = *ℱ*. This simple example is revisited in the Appendix to give concrete illustrations of some of the abstract measure-theoretic quantities deployed later in the paper.

That it might be advantageous to represent incomplete information as a randomly stopped stochastic process was hinted at by Gill et al. (1997), and we too find the simple partition of the sample space provided by *ℱ_M_* to be both instructive and illuminating as to the nature of the missing-data problem. We stress again that *F_M_* is not a random object; *𝒟* = *ℱ_M_* is a fixed subsigma algebra of *ℱ*. The sigma algebra *𝒟* is strictly smaller than *ℱ*; that is, there are events in *ℱ* that are not elements of *𝒟*. In particular, {00, 01} ∉ *𝒟* and so *𝒴* ⊈ *𝒟*. Equivalent characterizations of the data sigma algebra are possible, such as *𝒟* = *σ*(*M*, *Y*_1_, …, *Y_M_*), but do not provide such immediate and straightforward conditions for assessing data-measurability as are available once we realize that *𝒟* is a stopping-time sigma algebra.

### 3.2 Likelihood factorization

In this section, we describe sufficient conditions for factorization of a likelihood ratio. Gill et al. (1997) and Lu & Copas (2004) investigated when related conditions are also necessary, in the latter case within parametric families of measures. We continue with just the measures *P* and *Q*, but, whether comparing many models or just two, it is only when frequentist properties or procedures are of interest that we need concern ourselves with the actual data-generating mechanism. In our pure likelihood context, *P* and *Q* may be any pair of measures defined on (Ω,*ℱ*), and neither is assumed to be the true data-generating measure (Seaman et al., 2013). This said, the relevance of statistical calculations is clearly enhanced when posited models are plausible reflections of reality, so we examine the evaluation of model assumptions in § 5.

The best available likelihood comparison of *P* and *Q* is (d*P*/d*Q*)|_*𝒟*_. However, it is customary to suppose that scientific interest in these measures focuses on their behaviour (d*P*/d*Q*)|_*𝒴*_ on *𝒴*, and that their conditional likelihood ratio given *𝒴*, i.e., (d*P*/d*Q*)|_*ℱ*|*𝒴*_ = (d*P*/d*Q*)|_*ℱ*_/(d*P*/d*Q*)|_*𝒴*_ (Hoffman-Jørgensen, 1994, p. 130), is secondary because it describes only the so-called missingness mechanism. Formally, we assume that scientific interest lies in a parameter *θ* : *𝒫* → Θ, where *𝒫* is a set of probability measures on (Ω,*ℱ*), and where *𝒴* is sufficient for *θ* in the sense that (d*P*/d*Q*)|_*𝒴*_ = 1 implies *θ*(*P*) = *θ*(*Q*) for any *P*, *Q* ∈ *𝒫*.

Because scientific interest is restricted to *θ* and because fully specifying the conditional likelihood ratio (d*P*/d*Q*)|_*ℱ*|*𝒴*_ can be difficult or inconvenient, we may choose instead to focus evidential comparisons on the marginal likelihood ratio (d*P*/d*Q*)|_*𝒴*_. Alas, this marginal likelihood ratio is not data-measurable in general, basically for the same reasons that d*P*/d*Q* is not data-measurable: because *𝒴* ⊈ *𝒟*. But by analogy with (1), where we express the likelihood ratio (d*P*/d*Q*)|_*𝒟*_ in terms of a conditional expectation of d*P*/d*Q* given the data, we could form a marginal, data-measurable likelihood ratio

$$Q\left({\frac{\text{d}P}{\text{d}Q}|}_{Y}\text{|}D\right).$$

A reasonable concern with such a procedure is that the value of (2) may somehow depend on the particular choice of missingness mechanism under *P* or *Q*, meaning that (2) was not a marginal likelihood ratio for *θ* after all. We show in Lemma 2 that within certain equivalence classes no such dependence exists, and hence within these equivalence classes (2) may justifiably be called a marginal likelihood ratio. This result is based on a foundational lemma strongly related to the exchange of *seeing* and *doing* in causal inference (Pearl, 2009), which we state first.

Lemma 1. *The likelihood ratio* d*P*/d*Q* *is* *𝒟*-*measurable if and only if* (d*P*/d*Q*)|_*ℱ*|*𝒟*_ = 1.

The proof is direct: d*P*/d*Q* is *𝒟*-measurable if and only if d*P*/d*Q* = (d*P*/d*Q*)|_*𝒟*_, and hence if and only if (d*P*/d*Q*)|_*ℱ*|*𝒟*_ = 1. The implication is that when d*P*/d*Q* is *𝒟*-measurable, expectations conditional on *𝒟* may be taken interchangeably with respect to *P* or *Q*; that is, *P*(*A* | *𝒟*) = *Q*(*A* | *𝒟*) for all *A* ∈ *ℱ*.

Lemma 2. *Write P* ~ *Q* (mod *𝒴*) *if and only if* (d*P*/d*Q*)|_*ℱ*|*𝒴*_ *is* *𝒟*-*measurable. Let P*, *P*′, *Q and Q*′*be measures all belonging to the same* ~-*equivalence class, and suppose that* (d*P*′/d*P*)|_*𝒴*_ = (d*Q*′/d*Q*)|_*𝒴*_ = 1. *Then*

$$Q\left({\frac{\text{d}P}{\text{d}Q}|}_{Y}|D\right)=Q^{\prime }\left({\frac{\text{d}P^{\prime }}{\text{d}Q^{\prime }}|}_{Y}|D\right)=1/P\left({\frac{\text{d}Q}{\text{d}P}|}_{Y}|D\right).$$

To prove the first equality, we have only to exchange (d*P*/d*Q*)|_*𝒴*_ for (d*P*′/d*Q*′)|_*𝒴*_; then, because (d*Q*′/d*Q*)|_*𝒴*_ = 1, which is clearly *𝒟*-measurable, and because (d*Q*′/d*Q*)|_*ℱ*|*𝒴*_ is *𝒟*-measurable since *Q* ~ *Q*′ (mod *𝒴*), it follows that d*Q*′/d*Q* is itself *𝒟*-measurable and that, by Lemma 1, expectations conditional on *𝒟* can be taken interchangeably with respect to *Q* or *Q*′. The proof of the second equality depends critically on *P* ~ *Q* (mod *𝒴*), but is otherwise unilluminating, so we omit it. Nevertheless, this second equality describes an important symmetry property exhibited by likelihood ratios, but not necessarily by modifications thereof like (2).

Within an equivalence class, Lemma 2 says that for any *P* and *P*′ that agree on *𝒴*, it does not matter whether we use *P* or *P*′ in the numerator of the marginal likelihood ratio (2) and, so long as *Q* and *Q*′ agree on *𝒴*, we may use either *Q* or *Q*′ in the denominator; the value of (2) is unchanged. Consequently, within an equivalence class, calling (2) a marginal likelihood ratio appears justifiable. Conversely, if *P* ≁ *Q* (mod *𝒴*), then (2) is not a marginal likelihood ratio: even the basic symmetry property of Lemma 2 fails to hold. So the restriction of likelihood comparisons to measures in the same equivalence class is important. Anticipating slightly, one equivalence class will be the set of measures under which data are missing at random.

Another reason that (2) may justifiably be called a marginal likelihood ratio is that within an equivalence class the usual likelihood ratio (d*P*/d*Q*)|_*𝒟*_ includes (2) as a multiplicative factor. This fact is so important that we state it as a lemma.

Lemma 3. *If P* ~ *Q* (mod *𝒴*), *then*

$${\frac{\text{d}P}{\text{d}Q}|}_{D}=Q\left({\frac{\text{d}P}{\text{d}Q}|}_{Y}|D\right)\times {\frac{\text{d}P}{\text{d}Q}|}_{ℱ|Y}.$$

The result follows directly from the fact that *P* ~ *Q* (mod *𝒴*) if and only if (d*P*/d*Q*)|_*ℱ*|*𝒴*_ is *𝒟*-measurable, and the latter can therefore be brought outside the conditional expectation.

Lemmas 2 and 3 show that, given a pair of measures defined only on the restricted space (Ω,*𝒴*), the marginal likelihood ratio (2) is a multiplicative factor in the likelihood ratio formed by extending these measures to the whole of (Ω,*ℱ*) and restricting to *𝒟*. Moreover, the marginal likelihood ratio (2) is invariant with respect to the particular way these extensions are constructed, always provided that such extended measures belong to the same ~-equivalence class. The downside to this attractive invariance is that we must choose an equivalence class within which to work. Moreover, because the equivalence classes are defined by a data-measurability condition, there can be no evidence in the data to support one choice over another (see Molenberghs et al., 2008). This choice must be made based on convenience, on meta-data considerations or, most usually, on a combination of both. We discuss convenience first, before moving on to meta-data considerations later in the paper.

It is fairly clear that computing the marginal likelihood ratio (2) would be reasonably straight-forward if in fact *𝒴* and *ℳ* were independent under *P* and *Q*, and this may be one reason to prefer equivalence classes in which such measures appear. In fact, the next lemma shows that all independence measures belong to the same equivalence class, within which the marginal likelihood ratio for *θ* has a very simple form.

Lemma 4. *Suppose that 𝒴 and ℳ are independent under P and Q*. *Then P* ~ *Q* (mod *𝒴*) *and*

$$Q\left({\frac{\text{d}P}{\text{d}Q}|}_{Y}|D\right)={\frac{\text{d}P}{\text{d}Q}|}_{D|ℳ}.$$

To prove this, we use the fact that independence of *𝒴* and *ℳ* under *P* and *Q* means that d*P*/d*Q* = (d*P*/d*Q*)|_*𝒴*_ × (d*P*/d*Q*)|_*ℳ*_, and hence that (d*P*/d*Q*)|_*ℱ*|*𝒴*_ = (d*P*/d*Q*)|_*ℳ*_, which is certainly *𝒟*-measurable. Conversely, (d*P*/d*Q*)|_*𝒴*_ = (d*P*/d*Q*)|_*ℱ*|*ℳ*_ under independence, so *Q*{(d*P*/d*Q*)|_*𝒴*_ | *𝒟*} = *Q*{(d*P*/d*Q*)|_*ℱ*_ | *𝒟*}*/*(d*P*/d*Q*)|_*ℳ*_, as required.

The equivalence class in which the independence measures lie is precisely the class of measures satisfying the condition Rubin (1976) calls missingness at random. Lemma 2 tells us that within this class, the marginal likelihood ratio depends only on the behaviour of *P* and *Q* on *𝒴*, so using measures under which *𝒴* and *ℳ* are independent may be a convenient way to compute it. Lemma 3 shows that the marginal likelihood ratio for *θ* is a factor in the full likelihood, and Lemma 4 gives us the form of the marginal likelihood ratio directly. We put all these pieces together in the following central result.

Theorem 1. *Let P*, *P*′, *Q and Q*′ *be measures such that* (d*P*/d*P*′)|_*𝒴*_ = (d*Q*/d*Q*′)|_*𝒴*_ = 1. *Suppose that 𝒴 and ℳ are independent under P*′ *and Q*′, *and further assume that both P* ~ *P*′ (mod *𝒴*) *and Q* ~ *Q*′ (mod *𝒴*). *Then*

$${\frac{\text{d}P}{\text{d}Q}|}_{D}={\frac{\text{d}P^{\prime }}{\text{d}Q^{\prime }}|}_{D|ℳ}\times {\frac{\text{d}P}{\text{d}Q}|}_{ℱ|Y}.$$

Independence measures *P*′ and *Q*′ exist because of the product structure on (Ω,*ℱ*). The proof depends on Lemma 4, from which we deduce that *P*′ ~ *Q*′ (mod *𝒴*), and therefore also *P* ~ *Q* (mod *𝒴*) by transitivity. Since *P* ~ *Q* (mod *𝒴*), Lemma 3 provides a factorization of the full likelihood ratio, and Lemma 4 gives us the simpler form of the first term, as required.

Reducing likelihood factorizations to questions of data-measurability is a very general idea, and in particular none of the current section depends in any way on monotonicity of missing data. Indeed, our general approach applies equally well in any setting where the structure of the observed data is not fixed in advance. Unobserved quantities need not be thought of as data, but could simply be latent variables, for example random effects. This is the perspective taken by Farewell et al. (2017). An appealing advantage of the theory developed in the present paper is that it can more easily be extended to cases where the range of *M* is uncountable (Commenges & Gegout-Petit, 2015, p. 14), and we explore some of these possibilities in § 5. Gill et al. (1997) provided an exactly analogous likelihood decomposition in the setting of coarsened data, and they too highlighted the simplification arising from the working assumption of independence, which Jacobsen & Keiding (1995) call a reference model.

So far we have shown that a working independence conditional likelihood ratio is the unique marginal likelihood ratio for the parameter of interest *θ* within a particular equivalence class, and that this in turn is a multiplicative factor in the full likelihood ratio. It remains to demonstrate that missingness at random fully characterizes this equivalence class, and to explore the suitability of this class for use in substantive problems of statistical inference.

### 3.3 Measurability

The factorization of Theorem 1 holds whenever the conditional likelihood ratios (d*P*/d*P*′)|_*ℱ*|*𝒴*_ and (d*Q*/d*Q*′)|_*ℱ*|*𝒴*_ are *𝒟*-measurable; we now discuss how to determine whether they are. We assume throughout this section that *P*, *P*′, *Q* and *Q*′ satisfy the conditions of Theorem 1; that is, *P* and *P*′ agree on *𝒴*, *Q* and *Q*′ agree on *𝒴*, and *𝒴* and *ℳ* are independent under *P*′ and *Q*′. We focus on the relationship between *P* and *P*′, with analogous considerations needed for *Q* and *Q*′. It seems likely that the conditional likelihood ratio (d*P*/d*P*′)|_*ℱ*|*𝒴*_ should be expressible in terms of conditional densities of *M* given *𝒴* under *P* and *P*′, and this we now assert to be the case. The proof of Lemma 5 is not especially illuminating, so we defer it to the Appendix.

Lemma 5. *Let P and P*′ *be measures on* (Ω,*ℱ*). *Define the stochastic processes* (*p_m_*) *and* ( $p_{m}^{\prime }$ ) *by p_m_* = *P*(*M* = *m* | *𝒴*) *and* $p_{m}^{\prime }=P^{\prime }(M=m|Y)$ . *Then* ${\left(\text{d}P/\text{d}P^{\prime }\right)|}_{ℱ|Y}=p_{M}/p_{M}^{\prime }$ , *the ratio of these stochastic processes evaluated at the random stopping time M*.

We deduce that *P* ~ *P*′ (mod *𝒴*) if both *p_M_* and $p_{M}^{\prime }$ are *𝒟*-measurable. We see immediately that the latter is always *𝒟*-measurable: because *𝒴* and *ℳ* are independent under *P*′, $p_{m}^{\prime }=P^{\prime }(M=m|Y)=P^{\prime }(M=m)$ , and hence the process ( $p_{m}^{\prime }$ ) is in fact a deterministic sequence. The value of $p_{M}^{\prime }$ then depends only on *M*, which is certainly *𝒟*-measurable.

Data-measurability of *p_M_* is more subtle and not guaranteed. Our next result introduces our definition of missingness at random, and proves that this condition on a measure *P* establishes that the corresponding *p_M_* is data-measurable.

Lemma 6. *Suppose that P*(*M* = *m* | *𝒴*) = *P*(*M* = *m* | *Y_m_*) *for every m*. *Then* (*p_m_*) *is adapted to* (*ℱ_m_*), *and p_M_ is ℱ_M_*-*measurable*.

The proof is not difficult. For all *m*, the condition *P*(*M* = *m* | *𝒴*) = *P*(*M* = *m* | *𝒴_m_*) ensures that *P*(*M* = *m* | *𝒴*) is *𝒴_m_*-measurable, i.e., (*p_m_*) is adapted to (*𝒴_m_*). Since *Y_m_* ⊆ *ℱ_m_*, the stochastic process (*p_m_*) must also be adapted to (*ℱ_m_*). Because *M* is an (*ℱ_m_*)-stopping time, standard stochastic process results allow us to conclude that *p_M_* is *ℱ_M_*-measurable, as required.

We make several comments. First, the condition of Lemma 6 is equivalent to the rigorous, everywhere version of missingness at random (Seaman et al., 2013), but is simpler to state: by conditioning on sigma algebras *𝒴_m_*, we equate random variables, not a large set of conditional probabilities. Second, it applies equally well to a random variable *Y* taking values in uncountable spaces; neither discretization (Seaman et al., 2013) nor conditioning on a set of measure zero is required. Third, there is a striking visual similarity of the condition *P*(*M* = *m* | *𝒴*) = *P*(*M* = *m* | *𝒴_m_*) for all *m* to the ubiquitous, informal missing-at-random definition *P*(*M* | *Y*) = *P*(*M* | *Y*_obs_) (Little & Rubin, 2002, p. 12). Interpreted literally, though, the two definitions have rather different meanings: our definition of missing at random refers to a sequence of fixed values *m*, and not to the random variables *M* and *Y*_obs_. The measure-theoretic perspective encourages us to distinguish sharply between the random variable *M* and a generic realized value *m*, while the *Y*_obs_ notation blurs this distinction. In our view, this blurring, and the apparent collapse of multiple conditions into one that results from it, leads to much of the confusion surrounding the *Y*_obs_ and *Y*_mis_ notation (Seaman et al., 2013). This is another reason we think it is helpful to instead understand missingness at random as an adaptedness condition.

We have shown that if *P*(*M* = *m* | *𝒴*) = *P*(*M* = *m* | *𝒴_m_*) for every *m*, then *p_M_* and (d*P*/d*P*′)|_*ℱ*|*𝒴*_ are *𝒟*-measurable, and hence *P* belongs to the same ~-equivalence class as the independence measures. If the same is true for the measure *Q*, then the conditions of Theorem 1 are met, and the likelihood factorization follows. We now proceed to extend this result to nonmonotone missing data and, ultimately, to more general forms of missingness.

## 4 Nonmonotone missing data

We turn now to the general case, where there need be no natural ordering of the components of *Y*; the observations may be obtained simultaneously or in an arbitrary order, and any possible subset of the variables may be observed. Despite this generality, remarkably few notational changes are needed from the ordered, monotone case; we simply reinterpret what we have written to this point in terms of stochastic processes indexed by sets (Molchanov, 2006, p. 334). Our subscript *m* becomes a set, so that if *m* = {1, 3, 4} then *Y_m_* = (*Y*_1_, *Y*_3_, *Y*_4_). The most important change from the monotone case is that we now understand *M* as a random subset of {1, …, *n*}, representing the subset of variables that are observed. There is no total ordering of the subsets of {1, …, *n*}, but we exploit the partial ordering given by set inclusion and interpret *l* ⩽ *m* as *l* ⊆ *m*, which describes a lattice of subsets on which stochastic processes may be defined. Once again, we stop observing *Y* at the random set *M* on this lattice, but now there are potentially multiple routes by which we may arrive at a given point. Just as before, however, we observe the values of all random variables *Y_m_* for which *m* ⩽ *M*, i.e., for which *m* ⊆ *M*.

We define *𝒴_m_* = *σ*(*Y_l_* : *l* ⩽ *m*), *ℳ_m_* = *σ*({*M* ⩽ *l*} : *l* ⩽ *m*) and *ℱ_m_* = *𝒴_m_* ∨ *ℳ_m_* just as in the monotone case, where now (*ℱ_m_*) is a set-indexed filtration. As before, *𝒟* = *ℱ_M_*, now a stopping-set sigma algebra. The definitions of the probability measures *P*, *P*′, *Q* and *Q*′ are unaltered, and likelihood factorization again boils down to *ℱ_M_*-measurability of *p_M_*, where *p_m_* = *P*(*M* = *m* | *𝒴*), with similar considerations needed for *Q*.The missing-at-random condition is unaltered and forms the premise of our central theorem, which we now state formally.

Theorem 2. *Let P*, *P*′, *Q and Q*′ *be measures such that* (d*P*/d*P*′)|_*𝒴*_ = (d*Q*/d*Q*′)|_*𝒴*_ = 1. *Suppose that 𝒴 and ℳ are independent under P*′ *and Q*′. *Further, assume that P*(*M* = *m* | *𝒴*) = *P*(*M* = *m* | *𝒴_m_*) *and Q*(*M* = *m* | *𝒴*) = *Q*(*M* = *m* | *𝒴_m_*) *for all m*. *Then P* ~ *P*′ ~ *Q*′ ~ *Q* (mod *Y), and*

$${\frac{\text{d}P}{\text{d}Q}|}_{D}={\frac{\text{d}P^{\prime }}{\text{d}Q^{\prime }}|}_{D|ℳ}\times {\frac{\text{d}P}{\text{d}Q}|}_{ℱ|Y}.$$

The proof is identical to that in the monotone case: adaptedness of the processes *P*(*M* = *m* | *𝒴*) and *Q*(*M* = *m* | *𝒴*) to the set-indexed filtration (*ℱ_m_*), and the fact that *M* is an (*ℱ_m_*)-stopping set ensures that *P* ~ *P*′ (mod *𝒴*) and *Q* ~ *Q*′ (mod *𝒴*). Even in this unordered setting, the stochastic process techniques provide us with a direct proof of data-measurability of the likelihood ratios (d*P*/d*P*′)|_*ℱ*|*𝒴*_ and (d*Q*/d*Q*′)|_*ℱ*|*𝒴*_. The final likelihood factorization follows from Theorem 1, which was already of sufficient generality to accommodate the set-indexed case.

The likelihood factorization depends on the adaptedness of stochastic processes, so it is worth considering our ability to assess this collection of conditions. The lattice structure implicit in this formulation is reminiscent of the randomized monotone missingness mechanisms of Robins & Gill (1997), wherein future observation can depend on previous measurements within the history of a particular branch. But, as noted by Robins & Gill (1997), more complicated dependence structures are also possible. It may sometimes be appropriate to adopt the missingness-at-random assumption openly, but uncritically, and to use its simple ~-equivalence class to compare likelihoods or conduct inference under this working assumption. Alternatively, perhaps with improved intuition about what it means for a process to be adapted to a set-indexed filtration, the plausibility of the missingness-at-random assumption may be directly and critically assessed even in cases of nonmonotone missing data. Failing this, a fastidious analyst must abandon the appealing generality of missing at random, and assess instead conditions that are stronger and more easily assessed, such as randomized monotone missingness (Robins & Gill, 1997) or stability (Farewell et al., 2017). In § 5 we give specific examples of such stronger conditions.

## 5 Extensions

### 5.1 Modified notation

We now offer three extensions to the classical setting of Rubin (1976), showing how stochastic process theory adapts naturally to situations where traditional approaches can be cumbersome.

In this more general setting, we align our notation with standard choices made in the study of stochastic processes. Let *Y* = (*Y_t_* : *t* ∈ *T*) be a stochastic process indexed by a set *T*, where the latter may be uncountable, but is equipped with a partial or total ordering. We observe *Y_t_* for all *t* ⩽ *τ*, where the observed random variable *τ* takes values in *T*; we do not observe any *Y_t_* for *t* ⩽̸ *τ*. The process *Y* is adapted to its natural filtration (*𝒴_t_*), and again *τ* is a stopping time or more general stopping object with respect to the filtration (*𝒯_t_*) generated by the process ({*τ* ⩽ *t*}). We assume that *ℱ* = *𝒴* ∨ *𝒯*, where *𝒴* = *Ｖ_t_ 𝒴_t_* and similarly *𝒯* = *Ｖ_t_ 𝒯_t_*. We define another filtration (*ℱ_t_*) by *ℱ_t_* = *𝒴_t_* ∨ *𝒯_t_*. The observed data are then given by the generalized stopping-time sigma algebra *𝒟* = *ℱ_τ_* = {*A* ∈ *ℱ* : *A* ∩ {*τ* ⩽ *t*} ∈ *ℱ_t_* for all *t*}.

In this more general setting, it may well be the case that *P*(*τ* = *t* | *𝒴*) = 0 for all possible values of *t*, for example because *τ* might be a continuous random variable. Consequently, a slightly modified version of the missing-at-random condition will be required. As in the proof of Lemma 5, we could still define the conditional law *μ* of *τ* satisfying *μ*(*D*, ·) = *P*{*τ* ^−1^(*D*) | *𝒴*} and conditional densities like *p_t_* = (d*μ*/d*ν*)(*t*, ·) with respect to some dominating measure *ν*, but to avoid these abstractions we work instead with more familiar objects *P_t_* = *P*(*τ* ⩽ *t* | *𝒴*) that are analogous to conditional cumulative distribution functions. Like (*p_t_* : *t* ∈ *T*), the collection (*P_t_* : *t* ∈ *T*) is a stochastic process on the partially ordered set *T*. Since (*p_t_*) is adapted if and only if (*P_t_*) is adapted, it then suffices to define missingness at random in general as follows.

Lemma 7. *If P*(*τ* ⩽ *t* | *𝒴*) = *P*(*τ* ⩽ *t* | *𝒴_t_*) *for every t*, *then* (*P_t_*) *is adapted to* (*ℱ_t_*), *and P* ~ *P*′ (mod *𝒴*) *for any measure P*′ *under which 𝒴 and ℳ are independent*.

### 5.2 Explanatory variables

We suppose there is now some fully observed covariate information *𝒳* ⊆ *𝒟* available and that our interest is in the conditional likelihood ratio (d*P*/d*Q*)|_*𝒟*|*𝒳*_, as might be the case in a regression modelling context. An immediate advantage of the use of sigma algebras is that we can simply absorb this covariate information into the two existing filtrations (*𝒴_t_*) and (*𝒯_t_*), and assume that for all *t* we have *𝒳* ⊆ *𝒴_t_* and *𝒳* ⊆ *𝒯_t_*. If *𝒫* = {*P_αβγ_* } is a family of models wherein *α*, *β* and *γ* respectively characterize behaviour on *𝒳*, regression coefficients and distributions of residuals, with *θ*(*P_αβγ_*) = *β*, then (*𝒴* | *𝒳*) is sufficient for *θ* in the sense that for any *P*, *Q* ∈ *𝒫*, (d*P*/d*Q*)|_*𝒴*|*𝒳*_ = 1 implies *θ*(*P*) = *θ*(*Q*).

Lemma 4 will now hold under the weaker condition that *𝒴* and *𝒯* are conditionally independent given *𝒳*. By incorporating *X* into the two existing filtrations, we arrange for the missing-atrandom condition to remain notationally unchanged: *P*(*τ* ⩽ *t* | *𝒴*) = *P*(*τ* ⩽ *t* | *Y_t_*) for all *t*, recalling that now *𝒳* ⊆ *𝒴_t_* for all *t*. Like the condition employed by Sweeting et al. (2010), this allows dependence of the missingness mechanism on covariates and, to the extent permitted by data-measurabililty, on *Y*. This could be called covariate-dependent missingness at random, but, as noted by Hedeker & Gibbons (1997), versions such as this should be carefully distinguished from similarly named alternatives, where missingness depends only on covariates and not on observed responses (Little, 1995). The relevant likelihood factorization is

$${\frac{\text{d}P}{\text{d}Q}|}_{D|X}={\frac{\text{d}P^{\prime }}{\text{d}Q^{\prime }}|}_{D|T}\times {\frac{\text{d}P}{\text{d}Q}|}_{ℱ|Y},$$

where *Q*{(d*P*/d*Q*)|_*𝒴*|*𝒳*_ | *𝒟*} = (d*P*′/d*Q*′)|_*𝒟*|*𝒯*_ is the marginal likelihood particular to this equivalence class, integrating over the missing data. Since *𝒳* ⊆ *𝒯*, this likelihood is also conditional on *𝒳*. Our insistence that *𝒳* ⊆ *𝒴* pays dividends not only in notational brevity, but also in ensuring that intuitively important terms such as (d*P*/d*Q*)|_*𝒴*|*𝒳*_ remain well-defined.

The ability to include such covariate information *𝒳* is hugely important in missing-data considerations and, more broadly, in matters of causal inference. While hardly ever a trivial exercise, building a set of always-available data *𝒳* such that the required adaptedness condition may plausibly be assumed to hold is sometimes more straightforward than exerting external control of the processes that lead to aspects of *Y* going unobserved.

### 5.3 Longitudinal data

Consider the case where *Y* = (*Y_u_*)*_u_* ⩾_0_ is a continuous-time stochastic process and *τ* ⊆ [0, ∞) describes the finite set on which *Y* is observed. Such a construction describes unbalanced longitudinal data (Diggle et al., 2002, p. 282), where each subject gives rise to a random number of observations and these may be recorded at arbitrary points in time. For example, an individual observed at *u* = 0.2, *u* = 0.5 and *u* = 0.8 would have *τ* = {0.2, 0.5, 0.8}. The corresponding filtration (*𝒴_t_* : *t* ∈ *T*) is indexed by the power set *T* = 2^[0,∞)^ and therefore not totally ordered; instead, we again have a partial ordering *s* ⩽ *t* of the sets *s* and *t* if and only if *s* ⊆ *t*, and thus *𝒴_s_* ⊆ *𝒴_t_*. The set *T* of all possible subsets of [0, ∞) is uncountable, so our modified version of the missing-at-random condition will be required: *P*(*τ* ⩽ *t* | *𝒴*) = *P*(*τ* ⩽ *t* | *𝒴_t_*) for all *t* ∈ *T*. An example set *t* ∈ *T* for which such probabilities might be nonzero is *t* = [0, 0.3] ∪ [0.4, 0.9], in which case *τ* = {0.2, 0.5, 0.8} ⩽ *t*.

To our knowledge, characterizations of missingness at random for general longitudinal data are rare, and it is worth considering again our ability to assess this condition. Especially in longitudinal settings, the causal processes that lead to *τ* taking on any particular value rarely operate by first obtaining all possible information *𝒴* about *Y* and then deciding what subset of this information to reveal. Nevertheless, our formulation of missingness at random implicitly invites us to evaluate directly whether each *P*(*τ* ⩽ *t* | *𝒴*) in fact depends only on *𝒴_t_*.

We suggest taking a dynamic approach, in which the stopping set *τ* is itself thought of as arising from a set-valued stochastic process (*τ_u_*)_*u*⩾0_, with the set *τ_u_* = *τ* ∩ [0, *u*] defined to be the observed subset of *τ* up to and including time *u*. At each time *u* we observe a set-valued increment d*τ_u_* in this process, where d*τ_u_* = *τ_u_* \ *τ*_*u*−_ and *τ*_*u*−_ = *τ* ∩ [0, *u*). When no observation is made at time *u*, the increment d*τ_u_* is the empty set; when we make an observation at time *u*, the increment is the singleton set {*u*}.

With this more dynamic perspective, we are in a better position to assess the plausibility of missingness at random. Consider, for example, a patient under a so-called doctor’s care regime, which Grüger et al. (1991) define to mean that future examination times are determined entirely on the basis of earlier observations. Under such a regime, for an arbitrary set *t* ∈ *T* we can decompose *P*(*τ* ⩽ *t* | *𝒴*) using the product integral

$$\underset{u\in [0,\infty )}{\prod^{\text{​}}}P\left(\text{d}\tau_{u}⩽t|\tau_{u-}⩽t,Y\right)$$

wherein d*τ_u_*, *t* and *τ*_*u*−_ are all sets. Whenever *u* ∈ *t*, the integrand is unity; elsewhere it specifies the instantaneous probability that no observation is made at time *u*, given *𝒴*, and given the fact that all observation times to date lie in the set *t*. But we have asserted that under the doctor’s care scenario, future observation times are determined only with reference to past observations *𝒴*_*τ*_*u*−__, which must be a subset of *𝒴_t_* since we are conditioning on the event *τ*_*u*−_ ⩽ *t*. Hence *P*(d*τ*_*u*_ ⩽ *t* | *τ*_*u*−_ *t*, *𝒴*) = *P*(d*τ*_*u*_ ⩽ *t* | *τ*_*u*−_ ⩽ *t*, *𝒴_t_*), and our decomposition of *P*(*τ* ⩽ *t* | *𝒴*) multiplies back up to give *P*(*τ* ⩽ *t* | *𝒴_t_*), as required to establish that missingness at random holds. Here *P*(d*τ*_*u*_ ⩽ *t* | *τ*_*u*−_ ⩽ *t*, *𝒴*) is a kind of intensity process, and our doctor’s care assumption is very like independent censoring (Andersen et al., 1996, p. 139).

In situations where factors outside the doctor’s control may influence the number and timing of observations, such considerations become more delicate. For instance, if imperfectly measured subject-specific quantities such as a participant’s overall health may influence both *Y* and *τ*, the missing-at-random condition will not in general be satisfied. Farewell et al. (2017) make use of causal directed acyclic graphs to help determine if the likelihood contribution of the random observation times *τ* may safely be ignored.

### 5.4 Coarsened observations

For general coarsening of observations (Heitjan & Rubin, 1991), we shall assume as usual that *𝒴* represents complete information about some random variable *Y*. As in the longitudinal data setting, *Y* need not be scalar or even finite-dimensional; however, we now make the associated stochastic process implicit and instead begin with a specific filtration (*𝒴_t_* : *t* ∈ *T*) of *𝒴* that, as *t* ranges over *T*, visits some or all of the possible sub-sigma algebras of *𝒴*. This filtration supplies a partial ordering on *T* through the definition *s* ⩽ *t* if and only if *𝒴_s_* ⊆ *𝒴_t_*, and specifies the various possible levels of coarsening with which we may gain information about *𝒴*.

Heitjan & Rubin (1991, § 4.4) describes an example of coarsening, where children’s ages are recorded to an unknown degree of precision. Ages may be rounded to the next lowest month, half year or full year, so that a child with a recorded age of 6 months may in fact be up to 11 months old. Let *Y* record the age of the child in months, rounded to the next lowest month. There are three possible sub-sigma algebras of *𝒴* = *σ*(*Y*), namely *𝒴*_12_ = *σ*({*Y* < 12}, {12 ⩽ *Y* < 24}, {24 ⩽ *Y* < 36}, …), *𝒴*_6_ = *σ*({*Y* < 6}, {6 *Y* < 12}, {12 ⩽ *Y* < 18}, …) and *𝒴*_1_ = *𝒴* = *σ*({*Y* = 1}, {*Y* = 2}, {*Y* = 3}, …), with *𝒴*_12_ ⊆ *𝒴*_6_ ⊆ *𝒴*_1_. Here *T* = {12, 6, 1}, so a child with a recorded age of 18 months has an associated *τ* = 6; while the age *Y* of the child could equal 18 exactly, all we know is that it lies in the set {18, …, 23}. Suppose, for argument’s sake, that ages are in fact recorded to the next lowest month until one year of age, the next lowest half year until two years of age, and the next lowest full year thereafter. This constitutes a coarsening-at-random mechanism, because *P*(*τ* = *t* | *𝒴*) = *P*(*τ* = *t* | *𝒴*_12_) for all *t*. Formulating coarsening in terms of sigma algebras neatly captures the spirit of set-valued variables introduced by Heitjan & Rubin (1991).

## 6 Discussion

Initially, our aim in this work was to provide a rigorous reinterpretation of the usual missingness-at-random formulation *P*(*M* | *Y*) = *P*(*M* | *Y*_obs_) for those who, like ourselves, worry about such things. We hope that the version in Lemma 6 fits this bill. Seaman et al. (2013) point out that, interpreted literally, the symbol *Y*_obs_ might even tell us the value of *M*, but in fact no logical information about *M* is contained in any *𝒴_m_*, nor indeed in *𝒴* itself: for each nonempty set *A* ∈ *𝒴*, the image *M*(*A*) of *A* under *M* is simply the set *M*(Ω) of all possible values of *M*.

We believe that our work may have pedagogical value. Although we have attempted to convey our enthusiasm for the formalism of sigma algebras, an exactly equivalent version *P*(*M* = *m* | *Y*) = *P*(*M* = *m* | *Y_m_*) for all sets *m* does not rely on this concept. Those encountering this definition for the first time should see that there are many constituent subconditions, one for each possible subset *m* ⊆ {1, …, *n*}, and that the conditioning object *Y_m_* varies with *m*.

Our adaptedness requirement will seem natural to those familiar with stochastic processes, and provides further links between censoring and missing data (Aalen, 2007, 2012). The implied change of measure to a working independence setting also has a causal flavour: in causal inference, data-measurability is the key to identifiability of causal estimands, and employing stochastic bases {Ω, *ℱ*, (*ℱ_t_*)} with causal interpretations seems to us a promising approach.

## Supplementary Material

Appendix
