# Supplementary material for: Missing at random: a stochastic process perspective
Source: Biometrika. Author manuscript; Available in PMC 2022 Feb 2. (PMC7612310; doi:10.1093/biomet/asab002)
Supplement: Appendix [file EMS140624-supplement-Appendix.pdf]

argument's sake, that ages are in fact recorded to the next lowest month until one year of age, the next lowest half year until two years of age, and the next lowest full year thereafter. This constitutes a coarsening-at-random mechanism, because $P(\tau = t \mid \mathcal{Y}) = P(\tau = t \mid \mathcal{Y}_{12})$ for all t. Formulating coarsening in terms of sigma algebras neatly captures the spirit of set-valued variables introduced by Heitjan & Rubin (1991).

6. DISCUSSION

Initially, our aim in this work was to provide a rigorous reinterpretation of the usual missingness-at-random formulation $P(M \mid Y) = P(M \mid Y_{\text{obs}})$ for those who, like ourselves, worry about such things. We hope that the version in Lemma 6 fits this bill. Seaman et al. (2013) point out that, interpreted literally, the symbol Y_{obs} might even tell us the value of M, but in fact no logical information about M is contained in any $\mathcal{Y}_m$, nor indeed in $\mathcal{Y}$ itself: for each nonempty set $A \in \mathcal{Y}$, the image $M(A)$ of A under M is simply the set $M(\Omega)$ of all possible values of M.

We believe that our work may have pedagogical value. Although we have attempted to convey our enthusiasm for the formalism of sigma algebras, an exactly equivalent version $P(M = m \mid Y) = P(M = m \mid Y_m)$ for all sets m does not rely on this concept. Those encountering this definition for the first time should see that there are many constituent subconditions, one for each possible subset $m \subseteq \{1, \ldots, n\}$, and that the conditioning object Y_m varies with m.

Our adaptedness requirement will seem natural to those familiar with stochastic processes, and provides further links between censoring and missing data (Aalen, 2007, 2012). The implied change of measure to a working independence setting also has a causal flavour: in causal inference, data-measurability is the key to identifiability of causal estimands, and employing stochastic bases $\{\Omega, \mathcal{F}, (\mathcal{F}_t)\}$ with causal interpretations seems to us a promising approach.

ACKNOWLEDGEMENT

Odd Aalen, Daniel Commenges, Vern Farewell and Robin Henderson gave valuable advice during the writing of this paper. Rhian Daniel acknowledges support from a Sir Henry Dale Fellowship jointly funded by the Wellcome Trust and the Royal Society. Shaun Seaman was funded by the Medical Research Council.

APPENDIX

Examples of measure-theoretic quantities

Consider the simple setting introduced in § 3.1, in which a single binary Y_1 goes unobserved if $M = 0$, but is observed if $M = 1$. Recall that we defined Ω to be the four-point set $\{00, 01, 10, 11\}$ and that $\mathcal{F} = 2^{\Omega}$ was the power set of Ω. For a generic element $bc \in \Omega$, we let $Y_1(bc) = b$ and $M(bc) = c$.

In the case of finite sample spaces, we find it helpful to think of sigma algebras as partitioning the sample space into disjoint atoms, and have written the relevant sigma algebras to suggest this interpretation:

$$\begin{aligned}
\mathcal{Y}_0 &= \sigma(\{00, 10, 01, 11\}),\\
\mathcal{M}_0 = \mathcal{F}_0 = \mathcal{M}_1 = \mathcal{M} &= \sigma(\{00, 10\}, \{01, 11\}),\\
\mathcal{Y}_1 = \mathcal{Y} &= \sigma(\{00, 01\}, \{10, 11\}),\\
\mathcal{D} = \mathcal{F}_M &= \sigma(\{00, 10\}, \{01\}, \{11\}),\\
\mathcal{F}_1 = \mathcal{F} &= \sigma(\{00\}, \{01\}, \{10\}, \{11\}).
\end{aligned}$$

Downloaded from https://academic.oup.com/biomet/advance-article/doi/10.1093/biomet/asab002/6128501 by University of Cambridge user on 16 December 2021

For any such sigma algebra $\mathcal{G} \subseteq \mathcal{F}$, we may uniquely associate one of its corresponding atoms with each point in the sample space, so that $A_{\mathcal{G}}(\omega) \in \mathcal{G}$ is the atom containing ω. For example, $A_{\mathcal{Y}}(00) = \{00, 01\}$ and $A_{\mathcal{D}}(00) = \{00, 10\}$. Strictly speaking, these are only atoms under measures that assign them positive probability; we shall implicitly assume this to be the case for the measures that we go on to describe. For any such measures P and Q, we claim that

$$\left.\frac{\mathrm{d}P}{\mathrm{d}Q}\right|_{\mathcal{G}}(\omega) = \frac{P\{A_{\mathcal{G}}(\omega)\}}{Q\{A_{\mathcal{G}}(\omega)\}}$$

for all ω, as might reasonably be expected of a quantity we describe as a likelihood ratio. This can be verified by applying the measure-theoretic definition of the conditional expectation $Q(\mathrm{d}P/\mathrm{d}Q \mid \mathcal{G})$ to the constituent atoms $A_{\mathcal{G}}$ of each set $A \in \mathcal{G}$.

Let us now be more specific about the probability measures in question. We let $P(Y_1 = 1) = p$ and $P(M = 1 \mid Y_1 = y) = p_y$, say, with similar notation for Q, so that

$$P(\{bc\}) = p^b(1-p)^{1-b}p_b^c(1-p_b)^{1-c}, \quad Q(\{bc\}) = q^b(1-q)^{1-b}q_b^c(1-q_b)^{1-c}$$

for a generic element $bc \in \Omega$. This notation allows us to emphasize that it is really the relative success of p and q in explaining the distribution of Y_1 that is assumed to be of principal scientific interest. We have recycled some notation here; p_b and q_b are deterministic and distinct from the stochastic processes (p_m) and (q_m) used in the main body of the paper. It is now straightforward to write down $\mathrm{d}P/\mathrm{d}Q$:

$$\frac{\mathrm{d}P}{\mathrm{d}Q}(\omega) = \begin{cases} (1-p)(1-p_0)/(1-q)(1-q_0), & \omega = 00, \\ (1-p)p_0/(1-q)q_0, & \omega = 01, \\ p(1-p_1)/q(1-q_1), & \omega = 10, \\ pp_1/qq_1, & \omega = 11. \end{cases}$$

To evaluate its restriction to $\mathcal{D}$, we take each of the three atoms $A_{\mathcal{D}}$ of $\mathcal{D}$ in turn and get

$$\left.\frac{\mathrm{d}P}{\mathrm{d}Q}\right|_{\mathcal{D}}(\omega) = \begin{cases} \{(1-p)(1-p_0)+p(1-p_1)\}/\{(1-q)(1-q_0)+q(1-q_1)\}, & \omega \in \{00, 10\}, \\ (1-p)p_0/(1-q)q_0, & \omega = 01, \\ pp_1/qq_1, & \omega = 11. \end{cases}$$

Even more simply,

$$\left.\frac{\mathrm{d}P}{\mathrm{d}Q}\right|_{\mathcal{Y}}(\omega) = \begin{cases} (1-p)/(1-q), & \omega \in \{00, 01\}, \\ p/q, & \omega \in \{10, 11\}, \end{cases}$$

and

$$\left.\frac{\mathrm{d}P}{\mathrm{d}Q}\right|_{\mathcal{M}}(\omega) = \begin{cases} \{(1-p)(1-p_0)+p(1-p_1)\}/\{(1-q)(1-q_0)+q(1-q_1)\}, & \omega \in \{00, 10\}, \\ \{(1-p)p_0+pp_1\}/\{(1-q)q_0+qq_1\}, & \omega \in \{01, 11\}. \end{cases}$$

For a generic element $bc \in \Omega$, the measures P' and Q' of § 3.2 have corresponding probabilities

$$P'(\{bc\}) = p^b(1-p)^{1-b}\left(\frac{1}{2}\right)^c\left(1-\frac{1}{2}\right)^{1-c}, \quad Q'(\{bc\}) = q^b(1-q)^{1-b}\left(\frac{1}{2}\right)^c\left(1-\frac{1}{2}\right)^{1-c}$$

so that $(\mathrm{d}P/\mathrm{d}P')|_{\mathcal{Y}} = (\mathrm{d}Q/\mathrm{d}Q')|_{\mathcal{Y}} = 1$ as required, but now $\mathcal{Y}$ and $\mathcal{M}$ are independent under P' and Q'; the latter represent our working independence assumption. Replacing p_b and q_b by $1/2$ is an arbitrary

Downloaded from https://academic.oup.com/biomet/advance-article/doi/10.1093/biomet/asab002/6128501 by University of Cambridge user on 16 December 2021

choice; any two constant, positive probabilities will do. By inspecting the values taken by $(\mathrm{d}P/\mathrm{d}Q)|_{\mathcal{D}|\mathcal{M}}$ on its three atoms, or alternatively by applying Lemma 4, we see that

$$\left.\frac{\mathrm{d}P'}{\mathrm{d}Q'}\right|_{\mathcal{D}|\mathcal{M}}(\omega) = Q'\left(\left.\frac{\mathrm{d}P'}{\mathrm{d}Q'}\right|_{\mathcal{Y}}\right)(\omega) = \begin{cases} 1, & \omega \in \{00, 10\}, \\ (1-p)/(1-q), & \omega = 01, \\ p/q, & \omega = 11 \end{cases}$$

is our working independence conditional likelihood ratio, while

$$\left.\frac{\mathrm{d}P}{\mathrm{d}Q}\right|_{\mathcal{F}|\mathcal{Y}}(\omega) = \begin{cases} (1-p_0)/(1-q_0), & \omega = 00, \\ p_0/q_0, & \omega = 01, \\ (1-p_1)/(1-q_1), & \omega = 10, \\ p_1/q_1, & \omega = 11 \end{cases}$$

specifies the conditional likelihood ratio associated with the so-called missingness mechanism.

For the factorization of Theorem 1 to hold, we see that we require $\{(1-p)(1-p_0)+p(1-p_1)\}/\{(1-q)(1-q_0)+q(1-q_1)\}$ simultaneously to equal both $(1-p_0)/(1-q_0)$ and $(1-p_1)/(1-q_1)$, because $(\mathrm{d}P/\mathrm{d}Q)|_{\mathcal{D}}$ is constant on $\{00, 10\}$ while $(\mathrm{d}P/\mathrm{d}Q)|_{\mathcal{F}|\mathcal{Y}}$ need not be. There are two cases in which both equalities hold: either $p_0 = p_1$ and $q_0 = q_1$, or $p = q$ and $(1-p_0)(1-q_1) = (1-p_1)(1-q_0)$. In the former case, $\mathcal{Y}$ and $\mathcal{M}$ are independent, and hence the missing-at-random assumption is trivially satisfied. The latter case is uninteresting since we presumably set out to compare distinct p and q; nevertheless, it shows that missingness at random is not a necessary condition for the factorization to hold.

Proof of Lemma 5

Formally, M induces the conditional measure μ defined by $\mu(D, \omega) = P(M^{-1}(D) \mid \mathcal{Y})(\omega)$ for any $D \subseteq \{1, \ldots, n\}$ and $\omega \in \Omega$, with a similar definition for μ' in terms of P'. We label the conditional densities of μ and μ', taken with respect to counting measure ν, as $p_m = (\mathrm{d}\mu/\mathrm{d}\nu)(m, \cdot) = P(M = m \mid \mathcal{Y})$ and p'_m, defined equivalently. From their definitions, p_m and p'_m are random variables, and consequently both (p_m) and (p'_m) may be viewed as stochastic processes indexed by m. We now prove that p_M/p'_M, the ratio of these density processes evaluated at the random stopping time M, is indeed the desired conditional likelihood ratio $(\mathrm{d}P/\mathrm{d}P')|_{\mathcal{F}|\mathcal{Y}}$. We do so by showing that for any set $A \in \mathcal{F}$, p_M/p'_M converts the conditional probability of A under P', given $\mathcal{Y}$, to the corresponding conditional probability under P.

Let $A \in \mathcal{F}$, and recall that any such A equals BC for some $B \in \mathcal{Y}$ and $C \in \mathcal{M}$, so that $P'(A \times p_M/p'_M \mid \mathcal{Y}) = B \times P'(C \times p_M/p'_M \mid \mathcal{Y})$. But by definition of $\mathcal{M}$ we may write $C = M^{-1}(D) = D_M$, say, for some $D \subseteq \{1, \ldots, n\}$. A change of variables then allows us to write $P'(D_M \times p_M/p'_M \mid \mathcal{Y}) = \mu'(D \times p/p', \cdot)$. Now $\mathrm{d}\mu/\mathrm{d}\mu' = p/p'$, whence we have $P'(A \times p_M/p'_M \mid \mathcal{Y}) = B \times \mu(D, \cdot) = B \times P(C \mid \mathcal{Y}) = P(A \mid \mathcal{Y})$ as required.

REFERENCES

AALEN, O. O. (2007). Contribution to the discussion of ‘Longitudinal data with dropout: Objectives, assumptions and a proposal’ by P. J. Diggle, D. Farewell and R. Henderson. *Appl. Statist.* **56**, 538–9.

AALEN, O. O. (2012). Armitage lecture 2010: Understanding treatment effects: The value of integrating longitudinal data and survival analysis. *Statist. Med.* **31**, 1903–17.

ANDERSEN, P. K., BORGAN, O., GILL, R. D. & KEIDING, N. (1996). *Statistical Models Based on Counting Processes*. New York: Springer.

CHANG, J. T. & POLLARD, D. (1997). Conditioning as disintegration. *Statist. Neer.* **51**, 287–317.

COMMENGES, D. & GEGOUT-PETIT, A. (2015). Likelihood inference for incompletely observed stochastic processes: Ignorability conditions. *arXiv:* math/0507151v2.

DIGGLE, P., HEAGERTY, P., LIANG, K.-Y. & ZEGER, S. (2002). *Analysis of Longitudinal Data*. Oxford: Oxford University Press.

Downloaded from https://academic.oup.com/biomet/advance-article/doi/10.1093/biomet/asab002/6128501 by University of Cambridge user on 16 December 2021

Doretti, M., Geneletti, S. & Stanghellini, E. (2017). Missing data: A unified taxonomy guided by conditional independence. *Int. Statist. Rev.* **86**, 189–204.

Farewell, D. M., Huang, C. & Didelez, V. (2017). Ignorability for general longitudinal data. *Biometrika* **104**, 317–26.

Gill, R. D., van der Laan, M. J. & Robins, J. M. (1997). Coarsening at random: Characterizations, conjectures, counter-examples. In *Proc. First Seattle Sympos. Biostatistics*, D. Y. Lin & T. R. Fleming, eds., Lecture Notes in Statistics. New York: Springer, pp. 255–94.

Grüger, J., Kay, R. & Schumacher, M. (1991). The validity of inferences based on incomplete observations in disease state models. *Biometrics* **47**, 595–605.

Hedeker, D. & Gibbons, R. D. (1997). Application of random-effects pattern-mixture models for missing data in longitudinal studies. *Psychol. Meth.* **2**, 64–78.

Heitjan, D. F. & Rubin, D. B. (1991). Ignorability and coarse data. *Ann. Statist.* **19**, 2244–53.

Hoffman-Jørgensen, J. (1994). *Probability With a View Towards Statistics*, vol. II. Boca Raton, Florida: CRC Press.

Jacobsen, M. & Keiding, N. (1995). Coarsening at random in general sample spaces and random censoring in continuous time. *Ann. Statist.* **23**, 774–86.

Lin, X., Genest, C., Banks, D. L., Molenberghs, G., Scott, D. W. & Wang, J.-L., eds. (2014). *Past, Present, and Future of Statistical Science*. Boca Raton, Florida: Chapman and Hall/CRC.

Little, R. J. A. (1995). Modeling the drop-out mechanism in repeated-measures studies. *J. Am. Statist. Assoc.* **90**, 1112–21.

Little, R. J. A. & Rubin, D. B. (2002). *Statistical Analysis with Missing Data*. Hoboken, New Jersey: Wiley.

Lu, G. & Copas, J. B. (2004). Missing at random, likelihood ignorability and model completeness. *Ann. Statist.* **32**, 754–65.

Mealli, F. & Rubin, D. B. (2015). Clarifying missing at random and related definitions, and implications when coupled with exchangeability. *Biometrika* **102**, 995–1000.

Molchanov, I. (2006). *Theory of Random Sets*. New York: Springer.

Molenberghs, G., Beunckens, C., Sotto, C. & Kenward, M. G. (2008). Every missingness not at random model has a missingness at random counterpart with equal fit. *J. R. Statist. Soc.* B **70**, 371–88.

Pearl, J. (2009). *Causality*. Cambridge: Cambridge University Press.

Pollard, D. (2002). *A User's Guide to Measure Theoretic Probability*. Cambridge: Cambridge University Press.

Robins, J. M. & Gill, R. D. (1997). Non-response models for the analysis of non-monotone ignorable missing data. *Statist. Med.* **16**, 39–56.

Royall, R. (1997). *Statistical Evidence: A Likelihood Paradigm*. London: Routledge.

Rubin, D. B. (1976). Inference and missing data. *Biometrika* **63**, 581–92.

Seaman, S., Galati, J., Jackson, D. & Carlin, J. (2013). What is meant by 'missing at random'? *Statist. Sci.* **28**, 257–68.

Sweeting, M. J., Farewell, V. T. & Angelis, D. D. (2010). Multi-state Markov models for disease progression in the presence of informative examination times: An application to hepatitis C. *Statist. Med.* **29**, 1161–74.

[*Received on* 7 *December* 2018. *Editorial decision on* 23 *December* 2020]

Downloaded from https://academic.oup.com/biomet/advance-article/doi/10.1093/biomet/asab002/6128501 by University of Cambridge user on 16 December 2021
